# Unmasking of Olive Oil Adulteration Via a Multi-Sensor Platform

**DOI:** 10.3390/s150921660

**Published:** 2015-08-31

**Authors:** Marco Santonico, Simone Grasso, Francesco Genova, Alessandro Zompanti, Francesca Romana Parente, Giorgio Pennazza

**Affiliations:** 1 Center for Integrated Research—CIR, Unit of Electronics for Sensor Systems, Università Campus Bio-Medico, Via Alvaro del Portillo 21–Rome 00128, Italy; E-Mails: m.santonico@unicampus.it (M.S.); a.zompanti@unicampus.it (A.Z.); g.pennazza@unicampus.it (G.P.); 2 Center for Integrated Research—CIR, Unit of Food Science and Human Nutrition, Università Campus Bio-Medico, Via Alvaro del Portillo 21–Rome 00128, Italy; E-Mail: f.genova@unicampus.it; 3 Department of Industrial and Information Engineering and Economics, University of L’Aquila, Via Gronchi 18–L’Aquila 67100, Italy; E-Mail: francescaromana.parente@graduate.univaq.it

**Keywords:** olive oil authentication, olive oil adulteration, artificial sensorial system, food quality control, gas analysis, liquid analysis, BIONOTE (BIOsensor-based multisensorial system for mimicking Nose, Tongue and Eyes)

## Abstract

Methods for the chemical and sensorial evaluation of olive oil are frequently changed and tuned to oppose the increasingly sophisticated frauds. Although a plethora of promising alternatives has been developed, chromatographic techniques remain the more reliable yet, even at the expense of their related execution time and costs. In perspective of a continuous increment in the number of the analyses as a result of the global market, more rapid and effective methods to guarantee the safety of the olive oil trade are required. In this study, a novel artificial sensorial system, based on gas and liquid analysis, has been employed to deal with olive oil genuineness and authenticity issues. Despite these sensors having been widely used in the field of food science, the innovative electronic interface of the device is able to provide a higher reproducibility and sensitivity of the analysis. The multi-parametric platform demonstrated the capability to evaluate the organoleptic properties of extra-virgin olive oils as well as to highlight the presence of adulterants at blending concentrations usually not detectable through other methods.

## 1. Introduction

Olive oil is the most popular vegetable oil produced and consumed in Mediterranean countries. According to international standards [1], olive oils have to be obtained exclusively from the fruit of the olive tree (*Olea europaea*) using cold pressing techniques and in conditions that do not alter the organoleptic properties of the oil at all. Current European Union regulation [2] and the International Olive Committee (IOC) require olive oils to be graded in function of sensory assessment and three fundamental chemical parameters: free acidity, peroxide value, and UV absorbance [2]. By comparing oils scores with threshold values, these are classified as extra virgin olive oil (EVOO), virgin olive oil, and other low-quality olive oil typologies. Olive oil is a very complex matrix [3,4]. The main compounds are triacylglycerols and fatty acids contributing to 94%–96% of their total weight. However, triacylglycerols and fatty acid contents show a broad variability in olive oils chemical composition and this is largely dependent on both cultivar and geographical origin [5]. Recently, the authentication of products labeled as olive oil has become a fundamental issue for either commercial or health aspects [6,7]. In fact, the high price of olive oil and its increased popularity as a potential health food have made it an ideal target for frauds [8]. Common olive oil adulterations include accidental contaminations during production stages, deliberate mislabeling of less expensive oil categories and, more often, the admixtures of expensive olive oils with low quality oils. Although advances in knowledge and technology have undoubtedly led to greater success over frauds, even more complex forms of adulteration have been developed to invalidate the usefulness of official methods, thus leaving the authenticity verification still an unsolved matter [9]. Actually, no rapid and universal method exists that is officially recognized for all the authenticity issues [10]. Liquid and gas chromatographic techniques represent the elective methods for the authentication and characterization of individual olive oil compounds [11,12,13,14,15]. Nevertheless, these analytic verifications require valuable instrumentation and highly-qualified staff. All of these features together make authentication a time consuming and expensive process which is not applicable as routine analysis. In this context the BIONOTE (BIOsensor-based multisensorial system for mimicking Nose, Tongue and Eyes), a recently developed sensor platform [16], has been employed. The system, which embeds gas and liquid sensors having a common biologically-derived sensing interface, allows the simultaneous analysis of the vapor and liquid phase of the samples. As a consequence, the integrated multi-sensorial platform led different sensors to catch more comprehensive information which, in turn, requires a further elaboration through multivariate data analysis techniques. At the end of the analytical procedure, similarities and differences between the samples are highlighted. In this multi-parametric study, the correct discrimination of twelve EVOOs made up of dissimilar olive cultivars and having different geographical origin has been achieved. Furthermore, the high sensitivity and reproducibility of the analysis, which were guaranteed by the innovative electronic interface of the system, permitted the detection of fraudulent admixing of extraneous vegetable oils (pomace, soybean, sunflower seeds, and peanut oils) up to concentrations lower than 5%. These promising results altogether present BIONOTE as a rapid and economic tool for high-throughput screening analysis.

## 2. Materials & Methods

### 2.1. Oil Samples

Twelve EVOO samples, indicated in the paper as EVOO #1, #2, #3, and so on, were obtained from twelve different Italian orchards. Several characteristics of the oils are reported (Table 1). The commercial EVOO as well as the pomace, soybean, sunflower seeds, and peanut oils were bought at a local market.

**Table 1:** General EVOOs specifications.

Oil Sample	Geographical Origin	Year of Production	Oil Variety
EVOO #1	Laterba	2013/2014	Picoline
EVOO #2	Castellaneta	2013/2014	Leccino
EVOO #3	Laterba	2013/2014	Picoline (organic)
EVOO #4	Laterba	2013/2014	Arbequina (organic)
EVOO #5	Grottaglie and Crispiano	2013/2014	Picoline (50%), Nociara (35%), Leccino (15%)
EVOO #6	Crispiano	2013/2014	Leccino
EVOO #7	Grottaglie	2013/2014	Ogliarola
EVOO #8	Grottaglie	2013/2014	Picoline
EVOO #9	Grottaglie	2012/2013	Cellina di Nardò
EVOO #10	Laterba	2013/2014	Leccino
EVOO #11	Crispiano	2012/2013	Cellina di Nardò
EVOO #12	Crispiano	2012/2013	Cima di Melfi

### 2.2. Gas Analysis

Quartz Micro Balances (QMBs) with six functionalized piezoelectric sensors were used as transducers for the gas sensor array as already described [16]. In order to perform homogeneous gas measurements the following experimental set-up was used. A volume of 2 mL for each olive oil sample was placed in a 50 mL glass flask and kept for 10 min at room temperature to obtain an adequate headspace. Dehumidified reference air was pumped into the sensors chamber at a flow rate of 3 L/min for 10 min to desorb any volatile trace from sensors surface before every measure. Oil samples were analyzed five times, setting a sampling interval of 90 s.

### 2.3. Liquid Analysis

Electronic interface and sensors employed in the liquid analyses were the same described in Santonico *et al.* Cyclic voltammetry in the range from −1 to 1 V was performed using a triangular function at 10 mHz and a sampling interval of 1 second. Olive oil samples for liquid sensor analysis were prepared following the procedure reported below. Briefly, a volume of 1 mL of oil was poured into a tube with 3 mL of methanol 70% (*v*/*v*) and mixed vigorously for 1 min. The vial containing the oil-alcohol emulsion was centrifuged for 5 min at 1000 RCF and 4 °C to separate the two phases efficiently. Finally, the methanol phase was collected and stocked in ice up until the analysis.

### 2.4. Chemical Quality Control Analyses

Polyphenol content, free acidity, peroxide value, ∆K, and refractive index of olive oil samples have been assessed following the standard chemical testing methods [15]. Briefly, polyphenol content was evaluated by means of Folin-Ciocalteu method, according to the procedure reported by Singleton and Rossi [17]. Free acidity content [18] was evaluated, dissolving the samples in a mixture of equal parts by volume of ethyl ether (95%) and ethyl alcohol, thus titrating with an ethanolic solution of potassium hydroxide, using phenolphthalein as indicator. Results were reported as grams of oleic acid per 100 g of oil. To determine the peroxide value [19], oil samples were dissolved in chloroform and glacial acetic acid, then a solution of potassium iodide was added, leaving the mixture incubating for five minutes in the dark, and finally a titration of the generated iodine with a standard sodium thiosulphate solution, using starch solution as indicator, was performed. The peroxide value was expressed in terms of milliequivalents of active oxygen per kilogram able to oxidize potassium iodide under the operating conditions. The quality of the olive oils employed in this study was also assessed measuring the absorption bands between 200 and 300 nm [20]. Samples were dissolved in iso-octane to obtain 1% (*w*/*v*) solutions and the specific absorbance at 232 and 270 nm with reference to pure solvent was determined. These absorptions were expressed as specific extinctions, conventionally indicated by K. Finally, a ∆K value was calculated relating the maximum recorded absorbance at 270 nm against the absorption of surrounding spectral region (±4 nm). The refractometric index of olive oils was determined using the Abbé refractometer, paying attention to correct the recorded value on a temperature basis. Three independent parameter’s determinations were carried out for each test sample. All the reagents used in this study were of certified analytical quality.

### 2.5. Data Analysis

Multivariate data analysis: Principal Component Analysis (PCA) and Partial Least Square Discriminant Analysis (PLS-DA), was performed using PLS-Toolbox (Eigenvector Research Inc., Manson, WA, USA) in the Matlab Environment (The MathWorks, Natick, MA, USA). PLS-DA models have been calculated in order to detect EVO adulteration and investigate BIONOTE relevance to the chemical parameters.

## 3. Results

### 3.1. Olive Oil BIONOTE Characterization

**Figure 1:**
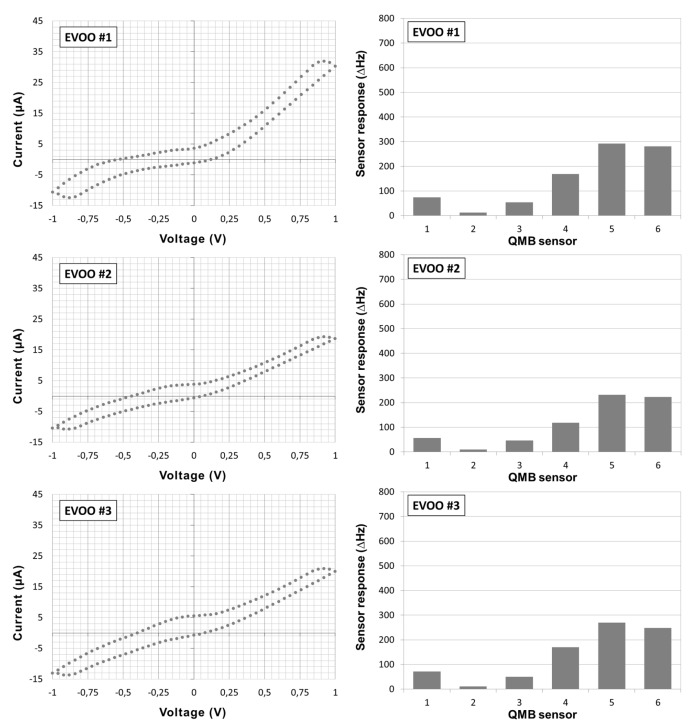

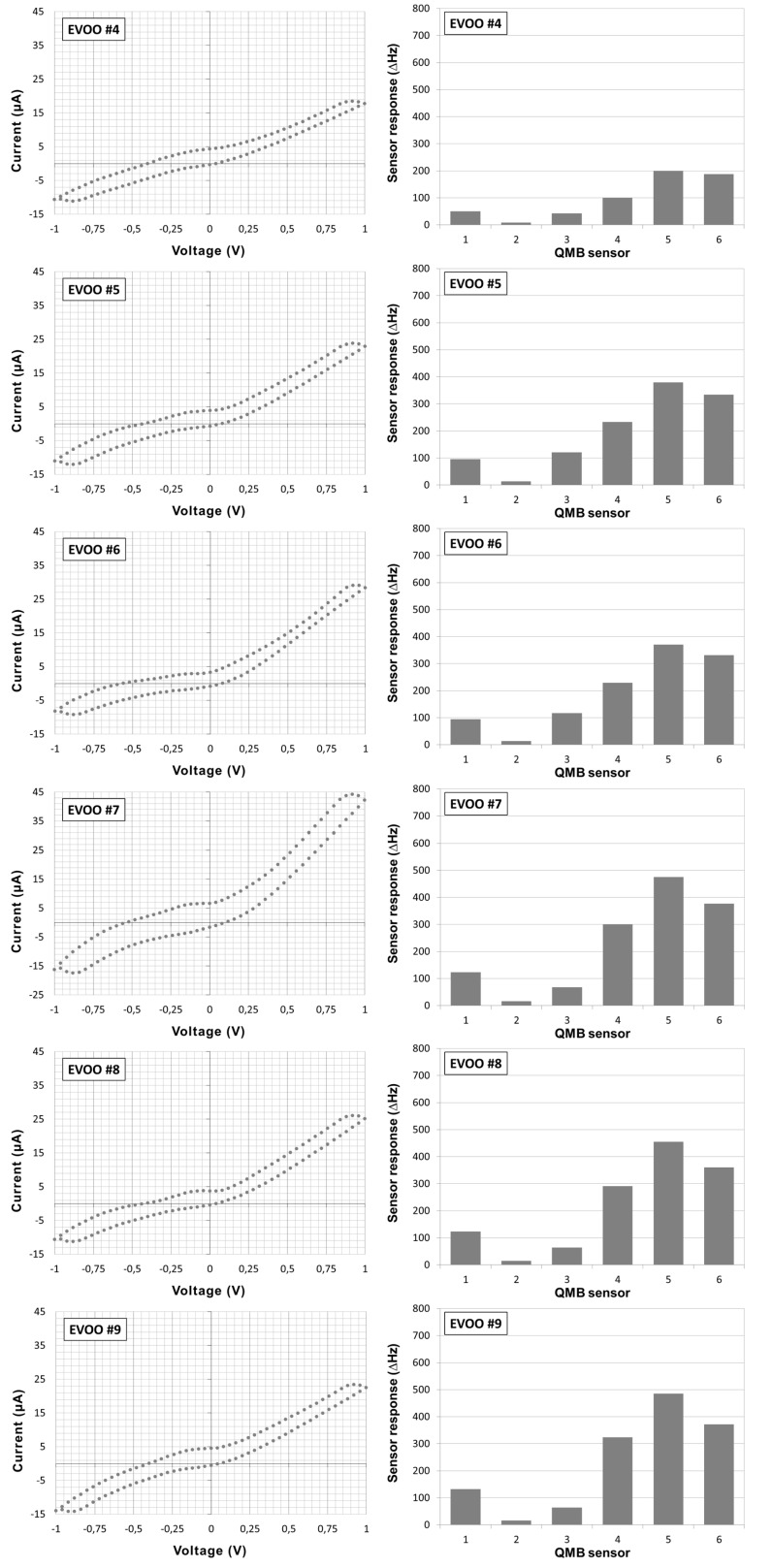

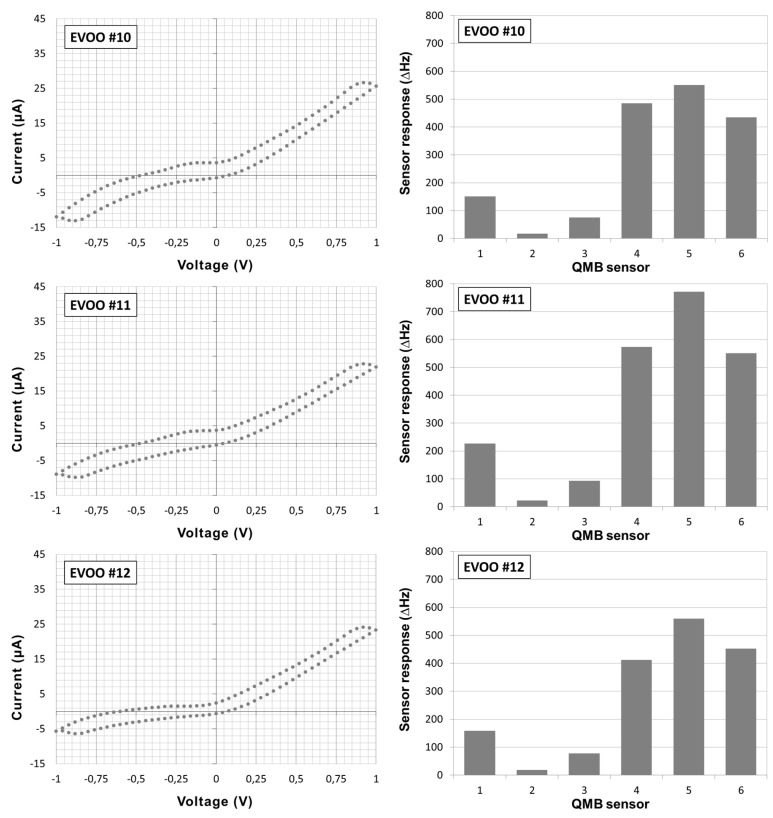
BIONOTE characterization of different EVOO samples. Liquid (**left panels**) and gas (**right panels**) fingerprints.

**Figure 2:**
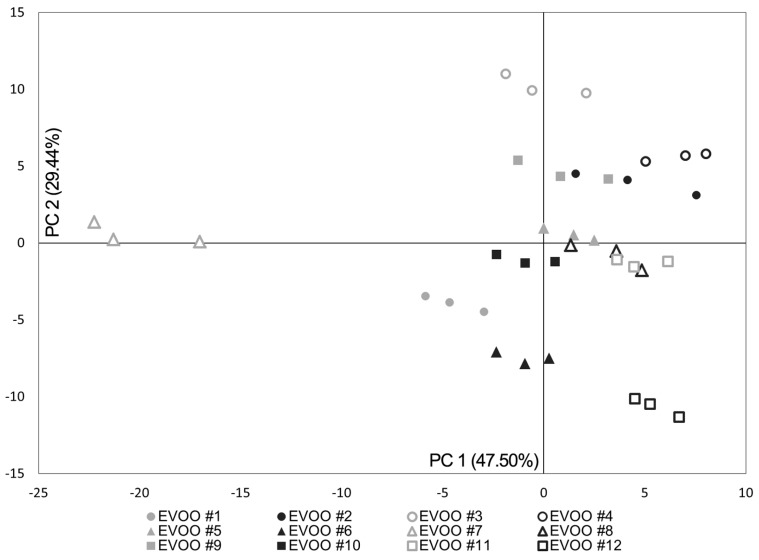
Score Plot of the first two principal components deriving from the data fusion of the BIONOTE liquid and gas sensors responses.

Twelve Italian EVOOs having different geographical origin and olive variety compositions have been characterized through the BIONOTE system, performing five measuring cycles each. Gas analysis was performed on EVOOs without any modification of the samples. Volatile compounds released in the system headspace at room temperature were characterized through their interaction with the functionalized sensors, resulting in a reproducible pattern response (Figure 1). Olive oil as such is not applicable for electrochemical analysis due to the absence of conductivity and the high viscosity of the media. Therefore, oil samples underwent liquid extraction with methanol and the deriving alcoholic fractions were analyzed by the liquid sensor (Figure 1). Cyclic voltammetry in the range from −1 to 1 V was performed using a triangular function at 10 mHz and a sampling interval of 1 second. By means of this setup, an array of 100 virtual sensor responses has been obtained from one physical sensor for each voltammetric measuring cycle. Finally, a data fusion of the information deriving from the last three measuring cycles of gas and liquid sensors was accomplished. The obtained data set has been evaluated by Principal Component Analysis (PCA) and the ability of the system to sharply discriminate the twelve EVOOs was demonstrated. The score plot of the first two Principal Components (PCs), accounting for 76.94% of the explained variance, is reported (Figure 2). Ten of the twelve oil samples clustered in three separate regions along the Principal Component 2 (PC2). EVOOs #1, #6, and #12 formed a group in the bottom part of the plane. EVOOs #5, #8, #10, and #11 distributed in a second area at the interception of the two PCs. EVOOs #2, #4, and #9 clustered in the upper portion of the plane (Figure 2). Nevertheless, within the groups almost every oil sample can be discriminated from the others along the Principal Component 1 (PC1). EVOOs #3 and #7 were distinguished from the rest of the analyzed samples by positioning at the upper end and at the left edge of the plane, respectively (Figure 2). Additionally, a Partial Least Square Discriminant Analysis (PLS-DA) model using the leave one out criterion has been calculated showing a correct classification rate of 100% for the twelve different EVOOs (five independent repetitions each).

### 3.2. Olive Oil Chemical Characterization

To assess the quality of the EVOOs, common chemical analyses were also performed. All the EVOOs got parameters satisfying the imposed normative limits, even though some slight differences between the samples were found (Table 2), thus supporting BIONOTE discrimination evidence. Free acidity and ∆K values were significantly lower than normative standard ones being, however, slightly different among each other. The refractive index of the twelve oil samples was almost the same, while the peroxide parameter showed the greatest variability. The obtained results confirmed the excellent quality of the oil samples, highlighting the absence (in terms of usual parameters) of significant differences between the EVOOs themselves.

**Table 2:** EVOO purity and quality characteristics according to the International Olive Council [1].

Oil Sample	Free Acidity (mg/100 g Oleic Acid)	Peroxide Value (mEq O_2_/Kg)	∆K	Refractive Index
EVOO #1	3.4 ± 0.1	15.0 ± 0.4	0.0020	1.469
EVOO #2	3.4 ± 0.1	12.2 ± 0.1	0.0045	1.468
EVOO #3	4.9 ± 0.2	6.0 ± 0.1	0.0065	1.468
EVOO #4	2.0 ± 0.1	6.9 ± 0.1	0.0015	1.467
EVOO #5	7.3 ± 0.1	8.7 ± 0.1	0.0015	1.468
EVOO #6	6.0 ± 0.1	9.5 ± 0.3	0.0005	1.467
EVOO #7	5.3 ± 0.1	7.2 ± 0.4	0.0030	1.467
EVOO #8	4.3 ± 0.2	18.1 ± 0.2	0.0045	1.468
EVOO #9	2.8 ± 0.1	9.9 ± 0.2	0.0035	1.468
EVOO #10	3.9 ± 0.1	13.5 ± 0.4	0.0015	1.467
EVOO #11	6.1 ± 0.2	9.4 ± 0.5	0.0030	1.467
EVOO #12	3.1 ± 0.2	9.9 ± 0.3	0.0160	1.467

### 3.3. Olive Oil Adulteration

**Figure 3:**
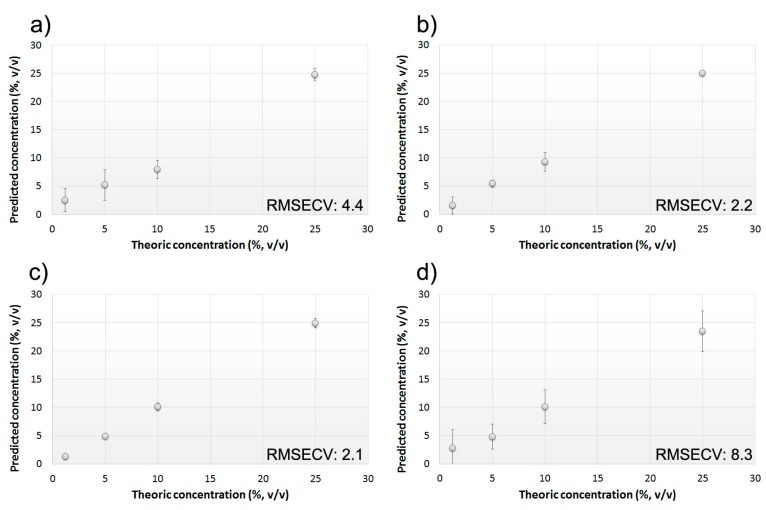
Calculated PLS-DA model for the prediction of contaminating oils concentration. Calibration model has been built using a commercial EVOO sophisticated with 0%–25% (*v*/*v*) of (**a**) soybean oil; (**b**) sunflower seeds oil; (**c**) peanut oil; and (**d**) pomace oil. RMSECV associated with the models are reported.

A commercial EVOO was bought at local market and mixed with four vegetable oils (pomace, soybean, sunflower seeds, and peanut oils) at different blending concentrations (1.25%, 5%, 10%, and 25% (*v*/*v*)). The prepared EVOO’s admixtures were characterized through the BIONOTE system, performing five measuring cycles each. Sophisticated EVOO samples were treated as already described (see Materials & Methods section) before being analyzed through either the liquid or the gas sensors. A comprehensive array containing the overall sensors’ responses was built for each EVOO sophistication independently and the collected data were further analyzed using multivariate data analysis techniques. The calculated PLS-DA models highlighted the ability of the system to distinguish an authentic EVOO from an adulterated one in all the tested cases, showing also a rather high degree of efficiency in the concentration discrimination (Figure 3). BIONOTE was able to predict the presence of contaminating lower-grade oils up to concentration values lower than 10% (*v*/*v*). The Root Mean Square Error in Cross Validation (RMSECV), using the Leave One Out criterion, was slightly different among the four kinds of sophistication. System performance was almost the same for the soybean, sunflower seeds, and peanut oils with RMSECV ranging from 2.1% to 4.4%, while the discrimination of the pomace oil sophistications resulted less precise accounting for an error of 8.3% (*v*/*v*) (Figure 3).

### 3.4. BIONOTE Relevance to the Chemical Parameters

**Figure 4:**
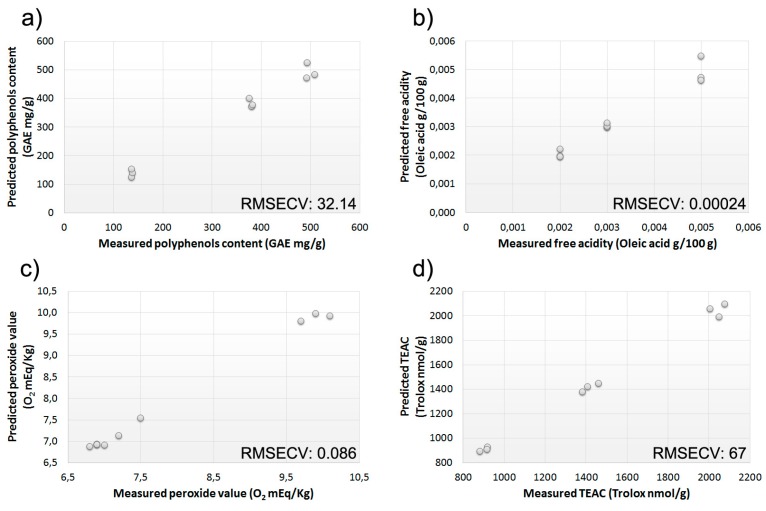
Measured *versus* predicted (PLS-DA model based on BIONOTE data) values of (**a**) polyphenols; (**b**) free acidity; (**c**) peroxide value; and (**d**) TEAC.

BIONOTE relevance to the measured chemical parameters have been investigated by calculating four different models to predict polyphenols content, free acidity, peroxide value, and TEAC on the gas and liquid sensor array data. The results obtained are very promising (see Figure 4, panel a: polyphenols; panel b: free acidity; panel c: peroxide value; and panel d: TEAC).

## 4. Discussion

Adulteration is a common problem usually related to high-value products. As a consequence of the fundamental role in the Mediterranean diet and the documented nutraceutical effect [6], EVOO represents a clear target for sophistication aimed to trade. According to recent studies, adulteration is becoming an escalating issue for olive oil in the market with consequences undermining the quality attributes of the product and sometimes even its safety consumption [21]. Although reliable and accurate analyses intended to guarantee olive oil quality in the broadest sense already exist, these are not routinely used. While chemical parameters as free acidity, peroxide value, ∆K, and refractive index are necessary to define if an olive oil fulfills the requirements to be labeled and marketed as EVOO, these constraints are not sufficient for authenticity verification in the most of cases [1,22]. Fraudulent olive oil admixtures are usually chemically corrected to meet international standards, thus requiring more complex analyses to be recognized as adulterations. Nevertheless, even when official analytical methods are applied to screen olive oil samples, olives’ biological differences, due to geographical origin and genetic aspects, sometimes generate problems to distinguish between sophistications and authentic EVOOs [23]. So far, numerous modern techniques have been proposed to support or replace official standard methods in the task of olive oil authentication [10,24,25,26,27,28]. However, those do not offer clear advantages yet, because their adulteration detection limits, being usually greater than 10% of contamination, are worse in comparison with chromatographic techniques’ ones. In this study, a novel system able to characterize EVOOs in terms of genuineness and authenticity has been presented. The BIONOTE platform takes advantage of either liquid and gas analysis to accomplish a multi-parametric characterization, giving comprehensive information about the sample [17]. The overall sensors’ responses are elaborated through multivariate data analysis techniques to highlight similarities and differences, resulting in a correct classification rate of 100%, even when similar EVOOs have been analyzed. Hence, BIONOTE showed the ability to discriminate between twelve Italian EVOOs originating from different Apulian neighboring olive tree orchards. The result highlighted the capability of BIONOTE not only to identify EVOOs against lower grade olive oils, but also to discriminate between EVOOs obtained from different olive cultivars. This is a notable outcome because this issue is usually addressed via more complex genetic approaches. The innovative electronic interface, providing to the system a higher reproducibility and sensitivity comparable to similar devices [29,30,31,32], allowed BIONOTE to be also successfully employed in the authenticity verification process, with admixtures percentage thresholds below the best levels reported by literature. BIONOTE was challenged with different kind of EVOO sophistications, covering concentrations lower than 10% (*v*/*v*), and in all cases it was able to distinguish authentic oil from an adulterated one. The system detected the presence of fraudulent admixing of extraneous vegetable oils (soybean, sunflower seeds and peanut oils) up to concentrations lower than 5%. However, when the pomace oil was used, system performance decreased. This discrepancy, leading to an increment of the detection limit to about 8%, could be probably explained by the shared origin between EVOO and pomace oil. Considering the demand of EVOO traceability and safety claimed by both producers and consumers, BIONOTE represents a potential solution. In fact, the BIONOTE system is able to address the EVOO authenticity issue focusing not only on the labeling control but also the genuineness of the oil, accounting for geographical origin and olive varieties composition at the same time.

## 5. Conclusions

Nowadays, global markets and international regulations have increased significantly the number of samples that require validation, raising the necessity of rapid analytical methods. In this context, BIONOTE could represent a real opportunity thanks to its reduced time of analysis. However, due to the profiling approach on which the system is based on, BIONOTE has not been intended to replace the high specificity of the official chromatographic methods. Hence, it is proposed as a rapid tool for preliminary high-throughput screening, aimed to detect samples that require further analytical verifications. This workflow has been designed to reduce the employment of high-value instrumentation and qualified personnel only to specific cases, thus decreasing the costs, while maintaining the elevated number of samples analyzed.
